# Small Airways: The “Silent Zone” of 2021 GINA Report?

**DOI:** 10.3389/fmed.2022.884679

**Published:** 2022-05-23

**Authors:** Marcello Cottini, Carlo Lombardi, Giovanni Passalacqua, Diego Bagnasco, Alvise Berti, Pasquale Comberiati, Gianluca Imeri, Massimo Landi, Enrico Heffler

**Affiliations:** ^1^Allergy and Pneumology Outpatient Clinic, Bergamo, Italy; ^2^Departmental Unit of Allergology, Immunology & Pulmonary Diseases, Fondazione Poliambulanza, Brescia, Italy; ^3^Allergy and Respiratory Diseases, IRCCS Policlinico San Martino, University of Genoa, Genova, Italy; ^4^Ospedale Santa Chiara and Department of Cellular, Computational and Integrative Biology (CIBIO), Thoracic Disease Research, University of Trento, Trento, Italy; ^5^Section of Pediatrics, Department of Clinical and Experimental Medicine, University of Pisa, Pisa, Italy; ^6^Respiratory Unit, Department of Medical Sciences, Papa Giovanni XXIII Hospital, University of Milan-Bergamo, Bergamo, Italy; ^7^Institute for Biomedical Research and Innovation, National Research Council, Palermo, Italy; ^8^Pediatric National Healthcare System, Turin, Italy; ^9^Department of Biomedical Sciences, Humanitas University, Milan, Italy; ^10^Personalized Medicine, Asthma and Allergy - IRCCS Humanitas Research Hospital - Rozzano, Milan, Italy

**Keywords:** small airways dysfunction, GINA report, bronchial asthma, asthma control, recommendations

## Abstract

Asthma is a chronic disease, affecting approximately 350 million people worldwide. Inflammation and remodeling in asthma involve the large airways, and it is now widely accepted that the small airways (those with an internal diameter <2 mm) are involved in the pathogenesis of asthma and are the major determinant of airflow obstruction in this disease. From a clinical perspective, small airways dysfunction (SAD) is associated with more severe bronchial hyperresponsiveness, worse asthma control and more exacerbations. Unlike the GOLD guidelines which, in their definition, identify COPD as a disease of the small airways, the Global Initiative for Asthma (GINA) guidelines do not refer to the prevalence and role of SAD in asthmatic patients. This decision seems surprising, given the growing body of compelling evidence accumulating pointing out the high prevalence of SAD in asthmatic patients and the importance of SAD in poor asthma control. Furthermore, and remarkably, SAD appears to possess the characteristics of a treatable pulmonary trait, making it certainly appealing for asthma control optimization and exacerbation rate reduction. In this mini-review article, we address the most recent evidence on the role of SAD on asthma control and critically review the possible inclusion of SAD among treatable pulmonary traits in international guidelines on asthma.

## Introduction

Asthma is a chronic condition affecting the airways, characterized by inflammatory infiltration and remodeling of the bronchial tree (1). Recently, small airways have been recognized as a major site of airflow limitation in both asthma and chronic obstructive pulmonary disease (2–5). According to the current Global Initiative for Asthma (GINA) guidelines, spirometry remains the method of choice in evaluating the respiratory function (6). However, conventional spirometry reflects mostly the variability and/or the reversibility of airway obstruction and is unable to sensitively evaluate small airways, becoming abnormal only when approximately 75% of small airways are obstructed (7). In recent years more specialized tests have been developed, which may better assess small-airways dysfunction (SAD). These tests are now moving from clinical research laboratories into routine clinical practice (8). Table 1 summarizes the techniques available for the assessment of small airways disease.

**Table 1:** Available techniques for the assessment of bronchial airways by size (small vs. large airways).

**Method**	**Small airway function**	**Large airway function**
Spirometry	FEF25–75%, FVC, FVC/SVC	FEV1, FEV1/FVC
Impulse oscillometry (IOS)	R5–R20, X5, ΔX5 in-esp, AX, Fres	R20
Single breath nitrogen washout (SBNW) or Multiple breath nitrogen washout (MBNW) test	Slope phase III, CV, CC, Sacin, Scond, LCI	
Body plethysmography	RV, RV/TLC	
High resolution computerized tomography (HRCT)	Air trapping, airway wall thickness	Airway wall thickness
Nuclear medicine (Scintigrapy, SPECT, PET)	Regional ventilation defects	
3He-MRI	Non-ventilated lung volume	
Bronchoscopy	Transbronchial biopsy, BAL	Endobronchial biopsy
Sputum induction	Late phase sputum	Early phase sputum

*AX, reactance area; Fres, resonant frequency; LCI, Lung Clearance Index; FEV1, forced expiratory volume in 1 s; FVC, forced vital capacity; R5, resistance at 5 Hz; R20, resistance at 20 Hz; RV, residual volume; Sacin and Scond, acinar and conductive airways ventilation heterogeneity; TLC, total lung capacity*.

In particular, impulse oscillometry (IOS) is an effort-independent modality based on the well-described forced oscillation technique (FOT) (9, 10) and has emerged as a method to measure pulmonary function in both children and adults (11, 12).

We previously reviewed the prevalence and negative impact of SAD on asthma control, without addressing the position of current international guidelines on the role of SAD in asthma (13). In recent years, several original studies and systematic reviews confirmed that SAD is associated with, among others, greater bronchial hyper-responsiveness, worse asthma control and severity, more nocturnal and exercise-induced symptoms, and a higher number of exacerbations (14–16). Nevertheless, unlike the GOLD guidelines (17) which, in their definition, identify COPD as a disease of the small airways, the Global Initiative for Asthma (GINA) guidelines do not refer to the prevalence and role of SAD in asthmatic patients (6). This decision seems surprising, given the growing body of compelling evidence accumulating pointing out the high prevalence of SAD in asthmatic patients and the importance of SAD in poor asthma control. Furthermore, and remarkably, SAD appears to possess the characteristics of a treatable pulmonary trait, making it certainly appealing for asthma control optimization and exacerbation rate reduction.

In this mini-review article, we address the most recent evidence on the role of SAD on asthma control and critically review the possible inclusion of SAD among treatable lung traits in international guidelines on asthma.

## Prevalence of Sad in Asthmatic Patients

Overall, the prevalence of SAD in patients with asthma is around 50–60% (18–21). In the ATLANTIS study, the largest multinational study showing the contribution of SAD to asthma severity, 91% of asthmatics was found to have SAD, defined as any abnormal physiological variable, and SAD was strongly present across all GINA severity stages (18). Several other cohort studies showed the prevalence of SAD as defined by impulse oscillometry (IOS) (19, 20, 22, 23). We found (19, 22) in a cohort of 400 community-managed patients with physician-diagnosed asthma an overall prevalence of SAD of 62% in all the GINA step classes (step 2 58.3%; step 3 60.9%; step 4 63.3%; step 5 78.6%). Abdo et al. (20) confirmed these data, finding an IOS-defined prevalence of SAD of 63% in 268 asthma patients, with a higher prevalence of SAD in higher severity GINA stages, i.e., steps 4–5. Table 2 shows the prevalence data of IOS-defined SAD in studies from recent years.

**Table 2:** Prevalence data of Impulse Oscillometry-defined small airways dysfunction (SAD) in recent studies.

**References**	**Tool of assessment**	**Measure**	**Prevalence of SAD**	
Anderson et al. (24)	Impulse oscillometry	R5-R20	BTS 2 65% BTS 3 64% BTS4 70%	
Postma et al. (18)	Impulse oscillometry	R5-R20	42%	
Cottini et al. (19, 22)	Impulse oscillometry	R5-R20	62%	GINA 2 58%
				GINA 3 61%
				GINA 4 63%
				GINA 5 78%
Abdo et al. (20)	Impulse oscillometry	R5-R20	63%	GINA 2–3 53%
				GINA 4–5 75%
Alfieri et al. (25)	Impulse oscillometry	R5-R20	48%	
Manoharan et al. (26)	Impulse oscillometry	R5-R20	42%	
Berti et al. (23)	Impulse oscillometry	R5-R20	84%	(Elderly asthmatic patients)

*R5, resistance at 5 Hz; R20, resistance at 20 Hz; R5-R20, the difference between R5 and R20*.

## Association of Sad With Specific Asthma Phenotypes and Poor Asthma Control

Regardless of its prevalence in asthma, identifying SAD is of particular importance since it is clearly associated with specific clinical features and worse asthma control (19). Ignoring these key aspects would reduce the chances of maintaining asthma control.

SAD was previously linked to some clinical phenotypes of patients, i.e., active smokers, elderly patients with long duration of asthma and presence of fixed airflow obstruction, patients with nocturnal and exercise-induced symptoms, severe/uncontrolled asthma (13–16). The limit of most of the available studies is that they analyze the association of single features with SAD, instead ofcomprehensively address multiple asthma features associated with SAD. In more recent studies, multivariable analyses, classification tree analysis and structural equation modeling indicated that exercise-induced symptoms, overweight/obesity, asthma-related nocturnal symptoms, older age, smoking, and T2 inflammation are strong independent predictors of SAD in patients with community-managed asthma (19, 20, 27). Furthermore, emerging evidence shows that small conducting airways are an important site of disease also in pediatric asthma and are affected from an early stage of the disease (28). These associations may be of help in distinguishing subjects with SAD among patients with asthma, especially when IOS cannot be performed.

Spirometry is the most commonly used procedure to assess pulmonary function and GINA Guidelines and the Expert Panel Report-3 Guidelines for the Diagnosis and Management of Asthma both stated that pulmonary function measures are weakly correlated with asthma symptoms (6, 29). This statement refers to the “standard” pulmonary function test, unable to sensitively evaluate small airways, despite a growing body of literature supporting the correlation of SAD with asthma features (19). For instance, IOS-measured SAD has been shown to be present in virtually all patients with uncontrolled asthma vs. one third with well-controlled asthma, and to correlate (i.e., as assessed by the value of the difference in the resistance at 5 and 20 Hz [R5-R20], the IOS physiological marker that most strongly correlates with SAD) with worst asthma control and higher GINA step categories (19). Of note, a very weak inverse correlation between the spirometry value FEF25-75 and R5-R20 has been observed (19).

**Figure 1:**
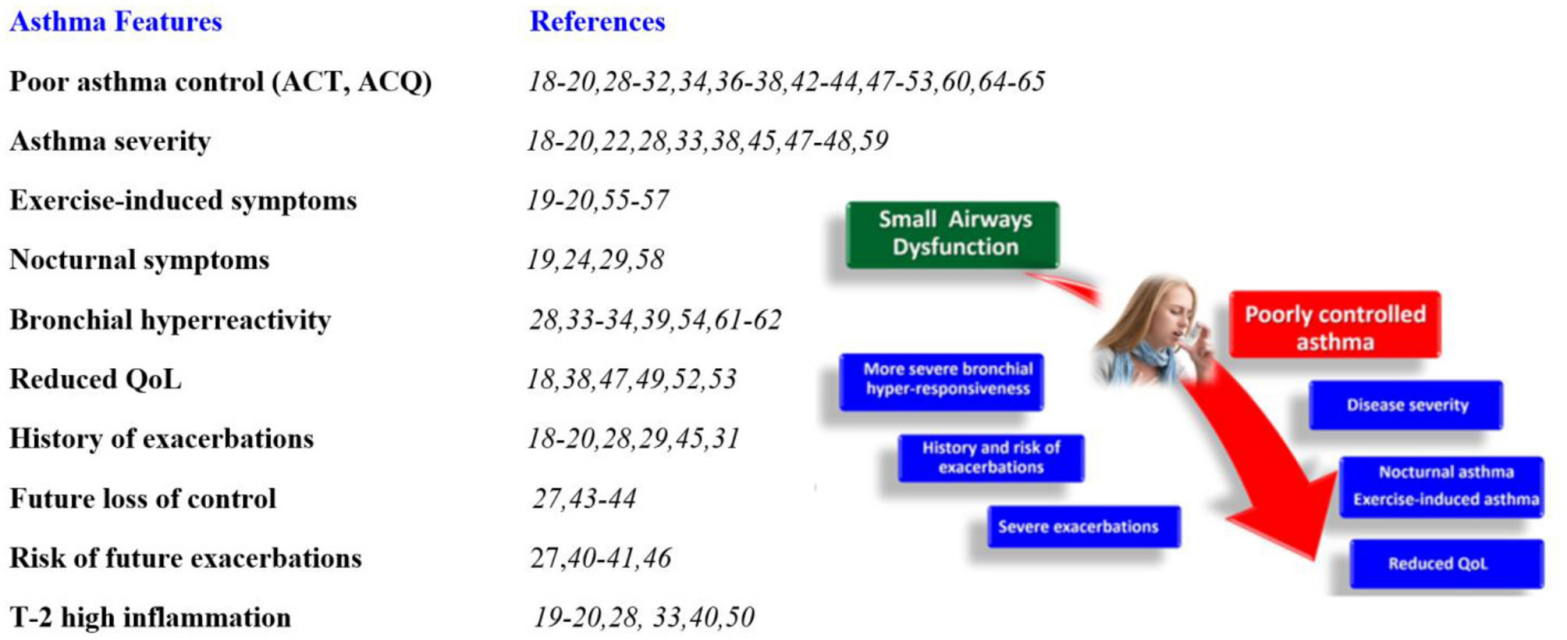
Association of small airways dysfunction with specific asthma features. ACT, asthma control test; ACQ, asthma control questionnaire; QoL, quality of life.

Similarly, Abdo et al. (20) recently showed that small airway dysfunction is strongly associated with poor control of the disease. In the ATLANTIS study (17), a SAD score (by both impulse oscillometry and spirometry) was significantly associated with asthma control, history of exacerbation, and disease severity. Kraft and colleagues very recently published the longitudinal one-year follow-up data of the ATLANTIS study, which showed that SAD (as measured by IOS, lung volumes, MBNW, and FEF25-75) was longitudinally associated with asthma control, exacerbations, and quality of life (30). In all of these studies, asthma control was intimately related to SAD and clinical phenotypes associated with SAD, and notably the IOS has better associations than spirometry, supporting the importance of using IOS and more modern tools in addition to spirometry. Studies supporting the relationship between asthma control and severity, asthma features, and SAD (18–28, 30–63) are summarized in Figure 1.

## Sad is a Treatable Pulmonary Trait

The term “precision medicine” usually refers to an emerging approach for disease treatment and prevention that takes into account individual variability in genes, environment, and lifestyle for each person (64). A new personalized approach, termed the “treatable traits” approach, has been suggested to address the limitations of the existing treatment strategies. IOS may be of great help to better characterize SAD as a “pulmonary treatable trait,” leading to a more targeted asthma management and more individualized patient care (64, 65). The importance of the peripheral airways in the pathophysiology and clinical manifestations of asthma makes them the intuitive target for long-term pharmacologic approaches (66, 67), i.e., for extra-fine formulations of bronchodilators and inhaled steroids (the mainstay treatment for COPD and asthma) and biologicals. Technological progress has allowed the development of new delivery systems and drug formulations designed to increase drug deposition and improve therapeutic efficiency, effectiveness, and drug safety (68–71). In real-life studies (72–75), extra-fine formulations (ICS and ICS/LABA) have shown significantly higher odds of achieving asthma control, even in small airway clinical phenotypes (76–78). Several real-life studies found an association between inhaled extra-fine ICS and ICS/LABA vs. standard particles size ICS or ICS/LABA and a reduction in airway resistance (18, 24).

Very intriguing, SAD may be modified by biologics; indeed, biologic therapies result not only in improvements in asthma control, OCS use, and exacerbation frequency but also in small airways function (54, 79–85).

## Conclusions

Despite the availability of effective therapies, a substantial proportion of asthmatics remain poorly controlled in real life. Given the clinical impact of SAD on asthma control, we believe that SAD should be actively searched as part of the daily management of patients with asthma. Since asthma control has been extensively proved to be linked with SAD, and SAD to be better assessed with IOS than conventional spirometry, we truly believe that IOS should complement spirometry as part of the routine diagnostic work-up of asthma patients in a *real-life* clinic setting. IOS-defined SAD can assist the clinician in understanding the risk of an asthma exacerbation in their patients along with routinely collected information on treatment intensity and asthma control. In clinical routine practice, the identification of SAD during the diagnostic work-up should influence clinicians on the treatment choice. Therefore, IOS may be of great help to better characterize SAD as a “pulmonary treatable trait,” leading to a more targeted asthma management and individualized patient care. Based on the above arguments, there appears to be an urgent need to implement the GINA recommendations with SAD, which is shown to be present in the majority of asthmatic patients and associated with worse disease control, helping to guide the therapeutic approach.

## Author Contributions

MC, CL, and GP contributed to conception and design of the study. AB organized the database. MC, CL, GP, PC, and ML wrote the first draft of the manuscript. EH wrote sections of the manuscript. All authors contributed to manuscript revision, read, and approved the submitted version.

## Conflict of Interest

The authors declare that the research was conducted in the absence of any commercial or financial relationships that could be construed as a potential conflict of interest.

## Publisher's Note

All claims expressed in this article are solely those of the authors and do not necessarily represent those of their affiliated organizations, or those of the publisher, the editors and the reviewers. Any product that may be evaluated in this article, or claim that may be made by its manufacturer, is not guaranteed or endorsed by the publisher.

## References

[1] Papi A Brightling C Pedersen SE Reddel HK. Asthma. Lancet. (2018) 391:783–800. 10.1016/S0140-6736(17)33311-1 29273246

[2] Braido F Scichilone N Lavorini F Usmani OS Dubuske L Boulet LP. Manifesto on small airway involvement and management in asthma and chronic obstructive pulmonary disease: an interasma (Global Asthma Association—GAA) and World Allergy Organization (WAO) document endorsed by Allergic Rhinitis and its Impact on Asthma (ARIA) and Global Allergy and Asthma European Network (GA2LEN). Asthma Res Pract. (2016) 2:12. 10.1186/s40733-016-0027-5 27965780 PMC5142416

[3] Mead J. The lung's “quiet zone”. N Engl J Med. (1970) 282:1318–9. 10.1056/NEJM197006042822311 5442364

[4] Hogg JC Macklem PT Thurlbeck WM. Site and nature of airway obstruction in chronic obstructive lung disease. N Engl J Med. (1968) 278:1355–6. 10.1056/NEJM196806202782501 5650164

[5] Burgel PR. The role of small airways in obstructive airway diseases. Eur Respir Rev. (2011) 20:23–33. 10.1183/09059180.00010410 21357889 PMC9487720

[6] GINA, report. Global Strategy for Asthma Management and Prevention. Available online at: https://ginasthma.org/gina-reports/ (accessed April 01, 2022).

[7] Cosio M Ghezzo H Hogg JC Corbin R Loveland M Dosman J. The relations between structural changes in small airways and pulmonary-function tests. N Engl J Med. (1978) 298:1277–81. 10.1056/NEJM197806082982303 651978

[8] Trinkmann F Watz H Herth FJF. Why do we still cling to spirometry for assessing small airway function? Eur Respir J. (2020) 56:2001071. 10.1183/13993003.01071-2020 32616553

[9] Dubois AB Brody AW Lewis DH Burgess BF Jr. Oscillation mechanics of lungs and chest in man. J Appl Physiol. (1956) 8:587–94. 10.1152/jappl.1956.8.6.587 13331841

[10] Cogswell JJ. Forced oscillation technique for determination of resistance to breathing in children. Arch Dis Child. (1973) 48:259–66. 10.1136/adc.48.4.259 4705931 PMC1648340

[11] Bednarek M Grabicki M Piorunek T Batura-Gabryel H. Current place of impulse oscillometry in the assessment of pulmonary diseases. Respir Med. (2020) 170:105952. 10.1016/j.rmed.2020.105952 32843158

[12] Kaminsky DA Simpson SJ Berger KI Calverley P de Melo PL Dandurand R. Clinical significance and applications of oscillometry. Eur Respir Rev. (2022) 31:210208. 10.1183/16000617.0208-2021 35140105 PMC9488764

[13] Cottini M Licini A Lombardi C Bagnasco D Comberiati P Berti A. Small airway dysfunction and poor asthma control: a dangerous liaison. Clin Mol Allergy. (2021) 19:7. 10.1186/s12948-021-00147-8 34051816 PMC8164746

[14] Cottini M Lombardi C Micheletto C. Small airway dysfunction and bronchial asthma control: the state of the art. Asthma Res Pract. (2015) 1:13. 10.1186/s40733-015-0013-3 27965766 PMC5142439

[15] van der Wiel E ten Hacken NH Postma DS van den Berge M. Small airways dysfunction associates with respiratory symptoms and clinical features of asthma: a systematic review. J Allergy Clin Immunol. (2013) 131:646–57. 10.1016/j.jaci.2012.12.1567 23380222

[16] Contoli M Bousquet J Fabbri LM Magnussen H Rabe KF Siafakas NM. The small airways and distal lung compartment in asthma and COPD: a time for reappraisal. Allergy. (2010) 65:141–51. 10.1111/j.1398-9995.2009.02242.x 19909298

[17] Global Initiative for Chronic Obstructive Lung Disease. Global Strategy for the Diagnosis, Management, Prevention of Chronic Obstructive Pulmonary Disease (2021 report) 2020. (2020). Available online at: https://goldcopd.org/wp-content/uploads/2020/11/GOLD-REPORT-2021-v1.1-25Nov20_WMV.pdf (accessed November 2, 2021).

[18] Postma DS Brightling C Baldi S Van den Berge M Fabbri LM Gagnatelli A. Exploring the relevance and extent of small airways dysfunction in asthma (ATLANTIS): baseline data from a prospective cohort study. Lancet Respir Med. (2019) 7:402–16. 10.1016/S2213-2600(19)30049-9 30876830

[19] Cottini M Licini A Lombardi C Berti A. Clinical characterization and predictors of IOS-defined small-airway dysfunction in asthma. J Allergy Clin Immunol Pract. (2020) 8:997–1004. 10.1016/j.jaip.2019.10.040 31726234

[20] Abdo M Trinkmann F Kirsten AM Pedersen F Herzmann C von Mutius E. Study Group. Small airway dysfunction links asthma severity with physical activity and symptom control. J Allergy Clin Immunol Pract. (2021) 9:3359–68. 10.1016/j.jaip.2021.04.035 33930619

[21] Usmani OS Singh D Spinola M Bizzi A Barnes PJ. The prevalence of small airways disease in adult asthma: a systematic literature review. Respir Med. (2016) 116:19–27. 10.1016/j.rmed.2016.05.006 27296816

[22] Cottini M Licini A Lombardi C Berti A. Prevalence and features of IOS-defined small airway disease across asthma severities. Respir Med. (2021) 176:106243. 10.1016/j.rmed.2020.106243 33253974

[23] Berti A Licini A Lombardi C Cottini M. Small airway dysfunction in elderly patient with asthma: a real life study. Eur Respir J. (2018) 52 (suppl 62):PA504. 10.1183/13993003.congress-2018.PA5045

[24] Anderson WJ Zajda E Lipworth BJ. Are we overlooking persistent small airways dysfunction in community-managed asthma? Ann Allergy Asthma Immunol. (2012) 109:185–189.e2 10.1016/j.anai.2012.06.022 22920073

[25] Alfieri V Aiello M Pisi R Tzani P Mariani E Marangio E. Small airway dysfunction is associated to excessive bronchoconstriction in asthmatic patients. Respir Res. (2014) 15:86. 10.1186/s12931-014-0086-1 25158694 PMC4243812

[26] Manoharan A Anderson WJ Lipworth J Ibrahim I Lipworth BJ. Small airway dysfunction is associated with poorer asthma control. Eur Respir J. (2014) 44:1353–5. 10.1183/09031936.00082314 25034570

[27] Abdo M Trinkmann F Kirsten AM Biller H Pedersen F Waschki B. Alliance study group. The relevance of small airway dysfunction in asthma with nocturnal symptoms. J Asthma Allergy. (2021) 14:897–905. 10.2147/JAA.S313572 34285516 PMC8286106

[28] Cottini M Lombardi C Berti A Comberiati P. Small-airway dysfunction in paediatric asthma. Curr Opin Allergy Clin Immunol. (2021) 21:128–34. 10.1097/ACI.0000000000000728 33620881

[29] National Heart Lung Blood Institute. Expert Panel Report 3: Guidelines for the Diagnosis and Management of Asthma. Available online at: https://www.nhlbi.nih.gov/health-topics/guidelines-for-diagnosis-management-of-asthma (accessed April 01, 2022).

[30] Kraft M Richardson M Hallmark B Billheimer D Van den Berge M Fabbri LM.; ATLANTIS study group. The role of small airway dysfunction in asthma control and exacerbations: a longitudinal, observational analysis using data from the ATLANTIS study. Lancet Respir Med. (2022) S2213-2600(21)00536-1. 10.1183/13993003.congress-2021.PA3722 35247313

[31] Riley CM Wenzel SE Castro M Erzurum SC Chung KF Fitzpatrick AM. Clinical implications of having reduced mid forced expiratory flow rates (FEF25-75), independently of FEV1, in adult patients with asthma. PLoS ONE. (2015) 10:e0145476. 10.1371/journal.pone.0145476 26717486 PMC4696666

[32] Chaiwong W Namwongprom S Liwsrisakun C Pothirat C. The roles of impulse oscillometry in detection of poorly controlled asthma in adults with normal spirometry. J Asthma. (2021) 6:1–37. 10.1183/13993003.congress-2020.2193 33356696

[33] Pisi R Tzani P Aiello M Martinelli E Marangio E Nicolini G. Allergy Asthma Proc. 2013Small airway dysfunction by impulse oscillometry in asthmatic patients with normal FEV1 values. Allergy Asthma Proc. (2013) 34:e14–20. 10.2500/aap.2013.34.3641 23406931

[34] Lipworth B Manoharan A Anderson W. Unlocking the quiet zone: the small airway asthma phenotype. Lancet Respir Med. (2014) 2:497–506. 10.1016/S2213-2600(14)70103-1 24899370

[35] Qin R An J Xie J Huang R Xie Y He L. FEF_25−75_% is a more sensitive measure reflecting airway dysfunction in patients with asthma: a comparison study using FEF_25−75_% and FEV_1_. J Allergy Clin Immunol Pract. (2021) 9:3649–3659.e6. 10.1016/j.jaip.2021.06.027 34214706

[36] Chiu HY Hsiao YH Su KC Lee YC Ko HK Perng DW. Small airway dysfunction by impulse oscillometry in symptomatic patients with preserved pulmonary function. J Allergy Clin Immunol Pract. (2020) 8:229–235.e3. 10.1016/j.jaip.2019.06.035 31299351

[37] Cottee AM Seccombe LM Thamrin C King GG Peters MJ Farah CS. Oscillometry and asthma control in patients with and without fixed airflow obstruction. J Allergy Clin Immunol Pract. (2022) S2213-2198(21)01453-7. 10.1016/j.jaip.2021.12.026. [Epub ahead of print]. 34979333

[38] Cottee AM Seccombe LM Thamrin C King GG Peters MJ Farah CS. Bronchodilator response assessed by the forced oscillation technique identifies poor asthma control with greater sensitivity than spirometry. Chest. (2020) 157:1435–41. 10.1016/j.chest.2019.12.035 31982392 PMC7268434

[39] Young HM Guo F Eddy RL Maksym G Parraga G. Oscillometry and pulmonary MRI measurements of ventilation heterogeneity in obstructive lung disease: relationship to quality of life and disease control. J Appl Physiol (1985). (2018) 125:73–85. 10.1152/japplphysiol.01031.2017 29543132

[40] Siroux V Boudier A Dolgopoloff M Chanoine S Bousquet J Gormand F. Forced mid-expiratory flow between 25% and 75% of forced vital capacity is associated with long-term persistence of asthma and poor asthma outcomes. J Allergy Clin Immunol. (2016) 137:1709–16.e6 10.1016/j.jaci.2015.10.029 26688518

[41] de Groot JC Amelink M de Nijs SB Plaat R Reitsma BH Storm H. Risk factors for frequent severe exacerbations in late-onset eosinophilic asthma. Am J Respir Crit Care Med. (2015) 192:899–902. 10.1164/rccm.201505-1003LE 26426787

[42] in 't Veen JC Beekman AJ Bel EH Sterk PJ. Recurrent exacerbations in severe asthma are associated with enhanced airway closure during stable episodes. Am J Respir Crit Care Med. (2000) 161:1902. 10.1164/ajrccm.161.6.9906075 10852764

[43] Bourdin A Paganin F Préfaut C Kieseler D Godard P Chanez P. Nitrogen washout slope in poorly controlled asthma. Allergy. (2006) 61:85–9. 10.1111/j.1398-9995.2006.00970.x 16364161

[44] Farah CS King GG Brown NJ Peters MJ Berend N Salome CM. Ventilation heterogeneity predicts asthma control in adults following inhaled corticosteroid dose titration. J Allergy Clin Immunol. (2012) 130:61–8. 10.1016/j.jaci.2012.02.015 22460065

[45] Farah CS King GG Brown NJ Downie SR Kermode JA Hardaker KM. The role of the small airways in the clinical expression of asthma in adults. J Allergy Clin Immunol. (2012) 129:381–7, 387.e. 10.1016/j.jaci.2011.11.017 22188824

[46] Busacker A Newell JD Jr Keefe T Hoffman EA Granroth JC Castro M. multivariate analysis of risk factors for the air-trapping asthmatic phenotype as measured by quantitative CT analysis. Chest. (2009) 135:48–56. 10.1378/chest.08-0049 18689585 PMC2849984

[47] Mummy DG Carey KJ Evans MD Denlinger LC Schiebler ML Sorkness RL. Ventilation defects on hyperpolarized helium-3 MRI in asthma are predictive of 2-year exacerbation frequency. J Allergy Clin Immunol. (2020) 146:831–39.e6. 10.1016/j.jaci.2020.02.029 32173351 PMC7487001

[48] Takeda T Oga T Niimi A Matsumoto H Ito I Yamaguchi M. Relationship between small airway function and health status, dyspnea and disease control in asthma. Respiration. (2010) 80:120–6 10.1159/000242113 19776554

[49] Jabbal S Manoharan A Lipworth J Lipworth B. Utility of impulse oscillometry in patients with moderate to severe persistent asthma. J Allergy Clin Immunol. (2016) 138:601–3. 10.1016/j.jaci.2015.12.1336 27016797

[50] Kuo CR Lipworth B. Airwave oscillometry and patient-reported outcomes in persistent asthma. Ann Allergy Asthma Immunol. (2020) 124:289–90. 10.1016/j.anai.2019.12.017 31904425

[51] Kuo CR Jabbal S Lipworth B. Is small airways dysfunction related to asthma control and type 2 inflammation? Ann Allergy Asthma Immunol. (2018) 121:631–2. 10.1016/j.anai.2018.08.009 30134181

[52] Heijkenskjöld Rentzhog C Janson C Berglund L Borres MP Nordvall L Alving K. Overall and peripheral lung function assessment by spirometry and forced oscillation technique in relation to asthma diagnosis and control. Clin Exp Allergy. (2017) 47:1546–155. 10.1111/cea.13035 28940832

[53] Bell AJ Foy BH Richardson M Singapuri A Mirkes E van den Berge M. Functional CT imaging for identification of the spatial determinants of small-airways disease in adults with asthma. J Allergy Clin Immunol. (2019) 144:83–93. 10.1016/j.jaci.2019.01.014 30682455

[54] Foy BH Soares M Bordas R Richardson M Bell A Singapuri A. Lung computational models and the role of the small airways in asthma. Am J Respir Crit Care Med. (2019) 200:982–91. 10.1164/rccm.201812-2322OC 31106566 PMC6794099

[55] Telenga ED van den Berge M Ten Hacken NH Riemersma RA van der Molen T Postma DS. Small airways in asthma: their independent contribution to the severity of hyperresponsiveness. Eur Respir J. (2013) 41:752–4. 10.1183/09031936.00170912 23456934

[56] Bahmer T Waschki B Schatz F Herzmann C Zabel P Kirsten AM. ERA-Study Group. Physical activity, airway resistance and small airway dysfunction in severe asthma. Eur Respir J. (2017) 49:1601827. 10.1183/13993003.01827-2016 28052957

[57] Mediano O Casitas R Villasante C Martínez-Cerón E Galera R Zamarrón E. Dynamic hyperinflation in patients with asthma and exercise-induced bronchoconstriction. Ann Allergy Asthma Immunol. (2017) 118:427–32. 10.1016/j.anai.2017.01.005 28214133

[58] Anderson SD. How does exercise cause asthma attacks? Curr Opin Allergy Clin Immunol. (2006) 6:37–42. 10.1097/01.all.0000199797.02423.78 16505610

[59] Kraft M Pak J Martin RJ Kaminsky D Irvin CG. Distal lung dysfunction at night in nocturnal asthma. Am J Respir Crit Care Med. (2001) 163:1551–6. 10.1164/ajrccm.163.7.2008013 11401872

[60] Shirai T Hirai K Gon Y Maruoka S Mizumura K Hikichi M. Forced oscillation technique may identify severe asthma. J Allergy Clin Immunol Pract. (2019) 7:2857–2860.e1 10.1016/j.jaip.2019.05.036 31231062

[61] Manoharan A Anderson WJ Lipworth J Lipworth BJ. Assessment of spirometry and impulse oscillometry in relation to asthma control. Lung. (2015) 193:47–51. 10.1007/s00408-014-9674-6 25516285

[62] Bao W Zhang X Yin J Han L Huang Z Bao L. Small-airway function variables in spirometry, fractional exhaled nitric oxide, and circulating eosinophils predicted airway hyperresponsiveness in patients with mild asthma. J Asthma Allergy. (2021) 14:415–26. 10.2147/JAA.S295345 33907426 PMC8071078

[63] Hou L Hao H Huang G Liu J Yu L Zhu L. The value of small airway function parameters and fractional exhaled nitric oxide for predicting positive methacholine challenge test in asthmatics of different ages with FEV1 ≥ 80% predicted. Clin Transl Allergy. (2021) 11:e12007. 10.1002/clt2.12007 33900045 PMC8099229

[64] Agustí A Bafadhel M Beasley R Bel EH Faner R Gibson PG. Precision medicine in airway diiseases: moving to clinical practice. Eur Respir J. (2017) 50:1701655. 10.1183/13993003.01655-2017 29051276

[65] Zimmermann SC Tonga KO Thamrin C. Dismantling airway disease with the use of new pul monary function indices. Eur Respir Rev. (2019) 28:180122. 10.1183/16000617.0122-2018 30918023 PMC9488242

[66] Santus P Radovanovic D Pecchiari M Ferrando M Tursi F Patella V. The relevance of targeting treatment to small airways in asthma and COPD. Respir Care. (2020) 65:1392–412. 10.4187/respcare.07237 32209703

[67] Lipworth B. Targeting the small airways asthma phenotype: if we can reach it, should we treat it? Ann Allergy Asthma Immunol. (2013) 110:233–9. 10.1016/j.anai.2013.02.009 23535085

[68] Scichilone N Benfante A Morandi L Bellini F Papi A. Impact of extrafine formulations of inhaled corticosteroids/long-acting beta-2 agonist combinations on patient-related outcomes in asthma and COPD. Patient Relat Outcome Meas. (2014) 5:153–162. 10.2147/PROM.S55276 25473323 PMC4251568

[69] Scichilone N Battaglia S Sorino C Paglino G Martino L Paternò A. Effects of extra-fine inhaled beclomethasone/formoterol on both large and small airways in asthma. Allergy. (2010) 65:897–902. 10.1111/j.1398-9995.2009.02306.x 20121764

[70] Usmani OS Baldi S Warren S Panni I Girardello L Rony F. Lung deposition of inhaled extrafine beclomethasone dipropionate/formoterol fumarate/glycopyrronium bromide in healthy volunteers and asthma: the STORM study. J Aerosol Med Pulm Drug Deliv. (2022). 10.1089/jamp.2021.0046. [Epub ahead of print]. 35128939 PMC9416540

[71] Lavorini F Pedersen S Usmani OS. Aerosol Drug Management Improvement Team. Dilemmas, confusion, and misconceptions related to small airways directed therapy. Chest. (2017) 151:1345–55. 10.1016/j.chest.2016.07.035 27522955

[72] Sonnappa S McQueen B Postma DS Martin RJ Roche N Grigg J. Extrafine versus fine inhaled corticosteroids in relation to asthma control: a systematic review and meta-analysis of observational real-life studies. J Allergy Clin Immunol Pract. (2018) 6:907–15.e7. 36. 10.1016/j.jaip.2017.07.032 28941668

[73] Müller V Gálffy G Eszes N Losonczy G Bizzi A Nicolini G. Asthma control in patients receiving inhaled corticosteroid and long-acting beta2-agonist fixed combinations: a real-life study comparing dry powder inhalers and a pressurized metered dose inhaler extrafine formulation. BMC Pulm Med. (2011) 11:40. 38. 10.1186/1471-2466-11-40 21762500 PMC3149024

[74] Allegra L Cremonesi G Girbino G Ingrassia E Marsico S Nicolini G. PRISMA (PRospectIve Study on asthMA control) Study Group. Real-life prospective study on asthma control in Italy: cross-sectional phase results. Respir Med. (2012) 106:205–14. 10.1016/j.rmed.2011.10.001 22035853

[75] Díaz-García R Flores-Ramírez G Ramírez-Oseguera RT. Efect of extrafne formulation of BDP/FF inhaler on asthma control, small airway function and airway infammation among Mexican asthmatic patients. A retrospective analysis. Respir Med. (2020) 165:105932. 10.1016/j.rmed.2020.105932 32308205

[76] Brusselle G Peché R Van den Brande P Verhulst A Hollanders W Bruhwyler J. Real-life effectiveness of extrafine beclometasone dipropionate/formoterol in adults with persistent asthma according to smoking status. Respir Med. (2012) 106:811–9. 10.1016/j.rmed.2012.01.010 22357130

[77] Marth K Spinola M Kisiel J Woergetter C Petrovic M Pohl W. Treatment response according to small airway phenotypes: a real-life observational study. Ther Adv Respir Dis. (2016) 10:200–10. 10.1177/1753465816642635 27060186 PMC5933612

[78] Carpagnano GE Scioscia G Lacedonia D Stornelli SR Quarato CMI Soccio P. Treatment response according to small airways disease status: the effects of high-strength extrafine pMDI beclomethasone dipropionate/formoterol fumarate in fixed dose combination in moderate uncontrolled asthmatic patients. Pulm Pharmacol Ther. (2020) 60:101879. 10.1016/j.pupt.2019.101879 31866498

[79] Farah CS Badal T Reed N Rogers PG King GG Thamrin C. Mepolizumab improves small airway function in severe eosinophilic asthma. Respir Med. (2019) 148:49–53. 10.1016/j.rmed.2019.01.016 30827474

[80] Sposato B Camiciottoli G Bacci E Scalese M Carpagnano GE Pelaia C. Mepolizumab efectiveness on small airway obstruction, corticosteroid sparing and maintenance therapy step-down in real life. Pulm Pharmacol Ther. (2020) 61:101899. 10.1016/j.pupt.2020.101899 31972327

[81] Shirai T Akamatsu T Hirai K Watanabe H Tamura K Kishimoto Y. Oscillometry improves earlier than spirometry after benralizumab initiation in severe asthma. Allergy. (2020) 75:2678–80 10.1111/all.14339 32339297

[82] Antonicelli L Tontini C Marchionni A Lucchetti B Garritani MS Bilò MB. Forced oscillation technique as method to document and monitor the efcacy of mepolizumab in treating severe eosinophilic asthma. Allergy. (2020) 75:433–78. 10.1111/all.13938 31166020

[83] Svenningsen S Eddy RL Kjarsgaard M Parraga G Nair P. Effects of anti-T2 biologic treatment on lung ventilation evaluated by MRI in adults with prednisone-dependent asthma. Chest. (2020) 158:1350–60. 10.1016/j.chest.2020.04.056 32428511

[84] Chan R RuiWen Kuo C Lipworth B. Real-life small airway outcomes in severe asthma patients receiving biologic therapies. J Allergy Clin Immunol Pract. (2021) 9:2907–9. 10.1016/j.jaip.2021.01.029 33545398

[85] Abdo M Watz H Veith V Kirsten AM Biller H Pedersen F. Small airway dysfunction as predictor and marker for clinical response to biological therapy in severe eosinophilic asthma: a longitudinal observational study. Respir Res. (2020) 21:278. 10.1186/s12931-020-01543-5 33087134 PMC7579879

